# Supplementary material for: The Landscape of Genetic Variation and Disease Risk in Romania: A Single-Center Study of Autosomal Recessive Carrier Frequencies and Molecular Variants
Source: Int J Mol Sci. 2025 Nov 11;26(22):10912. doi: 10.3390/ijms262210912 (PMC12652900; doi:10.3390/ijms262210912)
Supplement: Supplementary file 1 [file ijms-26-10912-s001.zip › Table S1.pdf]

Table A1. All genes and Pathogenic and Likely Pathogenic variants identified in the study

NO[1].	AR[2] GENES	NO[1]. INDIVIDUALS / GENE	GENE VARIANT	NO[1]. INDIVIDUALS / VARIANT
1	ABCB11	1	c.1445A>G (p.Asp482Gly)	1
2	ACAD9	5	c.728C>G (p.Thr243Arg)	1
			c.868G>A (p.Gly290Arg)	2
			c.1240C>T (p.Arg414Cys)	2
3	ACADM	11	c.244dupT (p.Trp82Leufs*23)	6
			c.614C>T (p.Ala205Val)	1
			c.617G>A (p.Arg206His)	1
			c.946-2A>C (Splice acceptor)	1
			c.985A>G, (p.Lys329Glu)	2
4	ACADVL	5	c.848T>C (p.Val283Ala)	1
			c.1077+2T>C (Splice donor)	1
			c.1097G>A (p.Arg366His)	1
			c.1700G>A (p.Arg567Gln)	2
5	ACSF3	2	c.1608G>A (p.Trp536*)	1
			c.1672C>T (p.Arg558Trp)	1
6	ADAMTS2	1	c.591dup (p.Leu198Alafs*7)	1
7	AGA	1	c.200_201delAG, (p.Glu67Alafs*3)	1
8	AGXT	1	c.508G>A (p.Gly170Arg)	1
9	AIRE	3	c.769C>T (p.Arg257*)	3
10	ALDOB	10	c.113-1_115del (Splice site)	2
			c.448G>C (p.Ala150Pro)	5
			c.524C>A (p.Ala175Asp)	2
			c.1005C>G (p.Asn335Lys)	1
11	ALMS1	2	c.4156dup (p.Thr1386Asnfs*15)	1
			c.11316_11319del (p.Glu3773Trpfs*18)	1
12	ALPL	3	c.395C>G (p.Ala132Gly)	1
			c.571G>A (p.Glu191Lys)	1
			c.395C>G (p.Ala132Gly)	1
13	AMT	1	c.217C>T (p.Arg73Cys)	1
14	ARSA	4	c.346C>T (p.Arg116*)	1
			c.542T>G (p.Ile181Ser)	1
			c.869G>A (p.Arg290His)	2
15	ARSB	1	c.1336+2T>G (Splice donor)	1
16	ASL	3	c.35G>A (p.Arg12Gln)	1
			c.857A>G (p.Gln286Arg)	2
17	ASS1	1	c.1087C>T (p.Arg363Trp)	1
18	ATM	2	c.1564_1565del (p.Glu522Ilefs*3)	1
			c.2250G>A (p.Lys750Lys)	1
19	ATP7A	1	c.4022G>A (p.Gly1341Asp)	1

20	ATP7B	17	c.19_20del (p.Gln7Aspfs*14)	2
			c.347T>C (p.Ile116Thr)	2
			c.1877G>C (p.Gly626Ala)	1
			c.2305A>G (p.Met769Val)	1
			c.2532delA (p.Val845Serfs*28)	1
			c.2605G>A (p.Gly869Arg)	1
			c.2817G>T (p.Trp939Cys)	4
			c.2906G>A (p.Arg969Gln)	1
			c.3207C>A (p.His1069Gln)	4
21	BBS1	5	c.1169T>G (p.Met390Arg)	5
22	BBS2	1	c.110del (p.Thr37Argfs*42)	1
23	BCKDHB	3	c.832G>A (p.Gly278Ser)	3
24	BCS1L	1	c.166C>T (p.Arg56*)	1
25	BLM	3	c.1642C>T (p.Gln548*)	2
			c.3013_3019+4del (Splice site)	1
26	BTD	37	c.1330G>C (p.Asp444His)	35
			c.1270G>C (p.Asp424His) = D424H	1
			c.1595C>T (p.Thr532Met) = T532M	1
27	CAPN3	5	c.550del (p.Thr184Argfs*36)	2
			c.956C>T (p.Pro319Leu)	1
			c.1342C>T (p.Arg448Cys)	1
			c.146G>A (p.Arg49His)	1
28	CBS	3	c.341C>T (p.Ala114Val)	3
29	CEP290	2	c.2991+1655A>G (Intronic)	1
			c.4522C>T (p.Arg1508*)	1
30	CERKL	2	c.375C>G (p.Cys125Trp)	1
			c.1042del (p.Ala348Profs*73)	1
31	CFTR	66	c.1210-7_1210-6del	1
			c.1210-11delinsGTG	1
			c.1210-34TG[11]T[5] (Intronic)	27
			c.1210-34TG[12]T[5] (Intronic)	3
			c.377G>A (p.Gly126Asp)	1
			c.1408A>G (p.Met470Val)= M470V	5
			c.1521_1523del (p.Phe508del)=F508del	19
			c.1624G>T (p.Gly542*)	1
			c.1807G>A (p.Val603Ile)	2
			c.2813T>G (p.Val938Gly)	1
			c.3472 C>G (Arg1158*)	2
			c.3846G>A (p.Trp1282*)	1
			c.3909C>G (p.Asn1303Lys)= N1303K	2
32	CHRNE	1	c.400_403dup (p.Ser135Trpfs*68)	1
33	CIITA	1	c.359-2A>G (Splice acceptor)	1
34	CLN3	1	c.46+1G>A (Splice donor)	1

35	CNGB3	3	c.819_826del (p.Arg274Valfs*13)	1
			c.1148del (p.Thr383Ilefs*13)	2
36	COL4A3	1	c.4825C>T (p.Arg1609*)	1
37	COL4A4	2	c.3973+1G>T (Splice donor)	1
			c.1321_1369+3del (Splice site)	1
38	COL7A1	6	c.425A>G (p.Lys142Arg)	2
			c.2005C>T (p.Arg669*)	1
			c.5086C>T p.Arg1696Cys	1
			c.7865G>A (p.Arg2622Gln)	1
			c.8038G>A (p.Gly2680Ser)	1
39	CPS1	1	c.2359C>T (p.Arg787*)	1
40	CPT2	4	c.338C>T (p.Ser113Leu)	4
41	CRB1	4	c.2308G>A (p.Gly770Ser)	3
			c.2843G>A (p.Cys948Tyr)	1
42	CYP11B2	6	Deletion (Exons 1-2)	1
			c.348delinsCT (p.Trp116Cysfs*21)	1
			c.554C>T (p.Thr185Ile)	2
			c.788T>A (p.Ile263Asn)	1
			c.1121G>A (p.Arg374Gln)	1
43	CYP17A1	1	c.1085G>A (p.Arg362His)	1
44	CYP21A2	31	c.92C>T (p.Pro31Leu)	2
			c.188A>T (p.His63Leu)	1
			c.293-13A/C>G (Intronic)	4
			c.332_339del (p.Gly111Valfs*21)	2
			c.844G>T (p.Val282Leu)	6
			c.955C>T (p.Gln319*)	5
			c.1069C>T (p.Arg357Trp)	1
			c.1360C>T (p.Pro454Ser)	10
45	CYP27A1	2	c.409C>T (p.Arg137Trp)	1
			c.1183C>T (p.Arg395Cys)	1
46	DCLRE1C	1	c.816T>A (p.Cys272*)	1
47	DHCR7	10	c.91C>T (p.Arg31Cys)	1
			c.452G>A (p.Trp151*)	5
			c.964-1G>C (Splice acceptor)	4
48	DLD	1	c.1123G>A (p.Glu375Lys)	1
49	DNAI1	2	*c.180G>A (Silent)*	2
50	DYSF	1	c.4726C>T p.Gln1576*	1
51	ELP1	3	c.741-2A>C (Splice acceptor)	2
			c.1289T>A (p.Leu430*)	1
52	ERCC6	2	c.1398-2A>G (Splice acceptor)	1
			c.3952_3953del (p.Arg1318Glyfs*12)	1
53	ESCO2	1	c.294_297del (p.Arg99Serfs*2)	1
54	ETFDH	1	c.652G>A (p.Asp218Asn)	1

55	EVC	7	c.673G>T (p.Gly225*)	1
			c.708dup (p.Ile237Tyrfs*5)	1
			c.919T>C (p.Ser307Pro)	5
56	EVC2	1	c.673G>T(p.Gly225*)	1
57	EYS	4	c.2137+1G>A (Splice donor)	2
			c.9036del (p.Leu3013Serfs*6)	2
58	FAH	1	c.1062+5G>A (Splice donor)	1
59	FANCA	2	Deletion (Exon 31)	1
			c.3788_3790delTCT (p.Phe1263del)	1
60	FANCC	3	c.844-1G>C (Splice acceptor)	3
61	FH	1	c.1431_1433dup (p.Lys477dup)	1
62	G6PD	5	c.563C>T (p.Ser188Phe)	4
			c.844G>C (p.Asp282His)	1
63	GAA	10	Deletion (Exon 18), c.-32-13T>G (Intronic)	2
			c.-32-13T>G (Intronic)	2
			c.1552-3C>G (Intronic)	2
			c.2066_2070dup (p.Ala691Serfs*7)	1
			c.2297A>G (p.Tyr766Cys)	1
			c.569G>A (p.Arg190His)	2
64	GALC	5	c.-119_-116del (Non-coding)	1
			c.908C>T (p.Ser303Phe)	3
			c.1204C>T (p.Arg402Trp)	1
65	GALT	32	c.-119_-116del (Non-coding)	25
			c.563A>G (p.Gln188Arg)	5
			c.855G>T (p.Lys285Asn)	2
66	GAMT	1	c.64dup (p.Ala22Gly fs*63)	1
67	GBA	5	c.115+1G>A (Splice donor)	1
			c.946C>T (p.Arg316Cys)	1
			c.1226A>G (p.Asn409Ser)	3
68	GBE1	4	c.1621A>G (p.Asn541Asp)	1
			c.2053-3358_2053-3350delins19 (Intronic)	1
			c.708G>C (p.Gln236His)	1
			c.986A>G (p.Tyr329Cys)	1
69	GCDH	2	c.1204C>T (p.Arg402Trp)	1
			c.892G>A (p.Ala298Thr)	1
70	GFM1	1	c.748C>T (p.Arg250Trp)	1
71	GJB2	35	c.-23+1G>A (Splice donor)	1
			c.35del (p.Gly12Valfs*2)	21
			c.313_326del (p.Lys105Glyfs*5)	1
			c.358_360del (p.Glu120del)	1
			c.370C>T (p.Gln124*)	2
			c.101T>C (p.Met34Thr)	4
			c.269T>C (p.Leu90Pro)	5

72	GLB1	1	c.202C>T (p.Arg68Trp)	1
73	GLDC	3	c.1896C>G (p.Tyr632*)	2
			c.2852C>A (p.Ser951Tyr)	1
74	GLE1	1	c.523_527del (p.Trp175Glyfs*6)	1
75	GNPTAB	1	c.1123C>T (p.Arg375*)	1
76	GNPTG	1	c.758C>A (p.Ser253*)	1
77	GP9	1	c.182A>G (p.Asn61Ser)	1
78	HAX1	1	c.214_217dup (p.Ile73Argfs*7)	1
79	HBA1	9	HBA1: Deletion (Entire coding sequence)	9
80	HBA2	1	HBA2: Deletion (Entire coding sequence)	1
81	HBB	3	c.-151C>T (Non-coding)	1
			c.155delC p.Pro52Leufs*10	1
			c.93-21G>A	1
82	HEXA	4	c.805G>A (p.Gly269Ser)	4
83	HEXB	1	c.1250C>T (p.Pro417Leu)	1
84	HFE	120	c.187C>G (p.His63Asp) §=H63D	100
			c.845G>A (p.Cys282Tyr) § =C282Y	19
85	HGD	1	c.16-1G>A (Splice acceptor)	1
86	HJV	3	c.959G>T (p.Gly320Val)	3
87	HLCS	1	Deletion (Exon 7)	1
88	HOGA1	1	c.700+5G>T (Intronic)	1
89	HPS3	1	c.998T>A (p.Leu333*)	1
90	HSD17B4	5	c.788del (p.Pro263Glnfs*2)	5
91	HYAL1	1	c.586_598del (p.Arg196Alafs*14)	1
92	IDUA	3	c.536C>T (p.Thr179Met)	1
			c.607G>A (p.Asp203Asn)	1
			c.1088_1089dup (p.Thr364Alafs*77)	1
93	LAMA2	4	Deletion (Exons 32-33)	1
			c.3607C>T (p.Gln1203*)	1
			c.3829C>T (p.Arg1277*)	1
			c.991A>T (p.Arg331*)	1
94	LAMA3	1	c.1144G>T (p.Glu382*)	1
95	LDLR	4	c.858C>A (p.Ser286Arg)	2
			c.1618G>A (p.Ala540Thr)	1
			c.2546C>A (p.Ser849*)	1
96	LIFR	2	c.1418del (p.Ser473Leufs*15)	1
			c.1789C>T (p.Arg597*)	1
97	LIPA	5	c.894G>A (Silent)	5
98	LOXHD1	2	c.3330_3333del (p.Asp1111*)	1
			c.4480C>T (p.Arg1494*)	1
99	MCCC1	3	c.1155A>C (p.Arg385Ser)	3
100	MCCC2	3	c.1015G>A (p.Val339Met)	3
101	MCOLN1	2	c.406-2A>G (Splice acceptor)	1

			c.1415_1419del (p.Asp472Valfs*134)	1
102	MEFV	4	c.2080A>G (p.Met694Val)	2
			c.2082G>A (p.Met694Ile)	2
103	MFSD8	1	c.754+2T>A Splice donor	1
104	MKS1	1	c.1476T>G(p.Cys492Trp)	1
105	MLC1	1	c.594_597delCTC A (p.Tyr198*)	1
106	MMAA	2	c.593_596del (p.Thr198Serfs*6)	2
107	MMAB	1	c.112_118delCAGG GCG (p.Gln38Trpfs*53)	1
108	MMACHC	2	c.271dup (p.Arg91Lysfs*14)	1
			c.481C>T (p.Arg161*)	1
109	MPL	2	c.1653del (p.Lys553Argfs*77)	1
			c.305G>C (p.Arg102Pro)	1
110	MPV17	2	c.71-2A>G (Splice acceptor)	1
			c.122G>A (p.Arg41Gln)	1
111	MTHFR	1	c.1753-18G>A (Intronic)	1
112	MTRR	1	c.1678_1681del (p.Glu560Asnfs*42)	1
113	MTTP	1	c.415C>T (p.Gln139*)	1
114	NEB	7	c.10872+2T>C*	1
			c.23989C>T (p.Arg7997*)	1
			c.24094C>T (p.Arg8032*)	5
115	NPC1	5	c.1114C>T (p.Arg372Trp)	1
			c.2536_2537del (p.Leu846Valfs*23)	2
			c.2861C>T (p.Ser954Leu)	1
			c.3019C>G (p.Pro1007Ala)	1
116	NPC2	1	c.441+1G>A Splice donor	1
117	NPHS2	3	c.868G>A (p.Val290Met)	3
118	NR2E3	6	c.119-2A>C (Splice acceptor)	4
			c.227G>A (p.Arg76Gln)	2
119	OAT	1	c.991C>T (p.Arg331*)	1
120	PAH	22	c.529G>C (p.Val177Leu)	1
			c.533A>G (p.Glu178Gly)	1
			c.545A>G (p.Glu182Gly)	1
			c.673C>A (p.Pro225Thr)	2
			c.844G>T (p.Val282Leu)	1
			c.1066-11G>A (Splice acceptor)	1
			c.1208C>T (p.Ala403Val)	1
			c.1222C>T (p.Arg408Trp)	5
			c.1315+1G>A (Splice donor)	1
			c.143T>C (p.Leu48Ser)	2
			c.734T>C (p.Val245Ala)	1
			c.844G>T (p.Val282Leu)	1
			c.898G>T (p.Ala300Ser)	4
121	PCCA	2	c.2041-2A>G (Splice acceptor	1

			c.183+1G>A (Splice donor)	1
122	PCCB	2	c.1540C>T (p.Arg514*)	2
123	PEX6	1	c.1314_1321del (p.Glu439Glyfs*3)	1
124	PEX10	1	c.4del (p.Ala2Profs*10)	1
125	PFKM	1	c.1761del (p.Ala588Leufs*29)	1
126	PKHD1	3	c.107C>T (p.Thr36Met)	2
			c.7916C>A (p.Ser2639*)	1
127	PMM2	1	c.124G>A (p.Gly42Arg)	1
128	PROP1	2	c.150del (p.Arg53Aspfs*112)	2
129	PTS	2	c.84-3C>G (Intronic)	1
			c.407A>T (p.Asp136Val)	1
130	PYGM	3	c.1561A>T (p.Lys521*)	1
			c.1805G>A (p.Arg602Gln)	2
131	RARS2	1	c.997C>G (p.Arg333Gly)	1
132	RMRP	2	n.239C>T (RNA change)	2
133	RPGRIP1L	1	c.3295-2A>G (Splice acceptor)	1
134	SEPSECS	1	c.811C>T (p.Arg271*)	1
135	SERPINA1	23	c.187C>T (p.Arg63Cys)	4
			c.194T>C (p.Leu65Pro)	3
			c.863A>T (p.Glu288Val) §	7
			c.1177C>T (p.Pro393Ser)	4
			c.1096G>A (p.Glu366Lys)	5
136	SGCG	1	Deletion (Exon 7)	1
137	SGSH	3	c.220C>T (p.Arg74Cys)	3
138	SLC12A3	5	c.457G>A (p.Val153Met)	1
			c.1180+1G>T (Splice donor)	1
			c.1742T>C (p.Met581Thr)	1
			c.1928C>T (p.Pro643Leu)	2
139	SLC22A5	5	c.136C>T (p.Pro46Ser)	1
			c.187C>T (p.Arg63Cys)	1
			c.844del* (p.Arg282Aspfs*14)	1
			c.364G>T (p.Asp122Tyr)	2
140	SLC26A2	7	c.-26+2T>C (Splice donor)	2
			c.1724del (p.Lys575Serfs*10)	2
			c.1957T>A (p.Cys653Ser)	3
141	SLC26A4	2	c.707T>C (p.Leu236Pro)	1
			c.1003T>C (p.Phe335Leu)	1
142	SMN1	20	Exon 7+8 deletion	14
			Gene deletion	6
143	SMPD1	3	c.820del (p.Met274Trpfs*25)	1
			c.[1268A>G];[?] (p.[His423Arg];[?])	1
			c.[1685T>A];[?] (p.[Met562Lys];[?])	1
144	TGM1	1	c.1469A>G (p.Asp490Gly)	1

145	TH	2	c.698G>A (p.Arg233His)	1
			c.707T>C (p.Leu236Pro)	1
146	TMEM216	1	c.218G>T (p.Arg73Leu)	1
147	TPP1	7	c.622C>T (p.Arg208*)	6
			c.1678_1679delCT	1
148	TRMU	1	Deletion (Exon 1)	1
149	TYMP	1	c.199C>T (p.Gln67*)	1
150	USH1C	2	c.1381G>T (p.Glu461*)	1
			c.1590+1G>T (Splice donor)	1
151	USH2A	14	c.2296T>C (p.Cys766Arg)	1
			c.2802T>G (p.Cys934Trp)	1
			c.6937G>T (p.Gly2313Cys)	1
			c.7524del (p.Arg2509Glyfs*19)	1
			c.8618T>G (p.Leu2873*)	1
			c.8682-9A>G (Intronic)	1
			c.10073G>A (p.Cys3358Tyr)	1
			c.11864G>A (p.Trp3955*)	2
			c.12268C>A (p.Pro4090Thr)	1
			c.12332C>T (p.Ser4111Phe)	2
			c.12569T>C (p.Val4190Ala)	1
			c.14803C>T (p.Arg4935*)	1
152	VPS13A	1	c.3769del (p.Ile1257Leufs*3)	1
153	VPS13B	4	Deletion (Exons 6-16)	1
			c.7524del (p.Arg2509Glyfs*19)	1
			c.8436+1G>A (Splice donor)	1
			c.9406-1G>T (Splice acceptor)	1
154	WNT10A	13	c.682T>A (p.Phe228Ile) §	13
155	XPA	1	c.772_785del (p.Arg258Tyrfs*5)	1
156	XPC	1	c.1A>G (p.Met1?)	1
**NO[1].**	**AD[3] GENES**	**NO[1]. INDIVIDUALS / GENE**	**GENE VARIANT**	**NO[1]. INDIVIDUALS / VARIANT**
1	F2	18	c.*97G>A (Non-coding) §	17
			c.187C>G (p.His63Asp) §	1
2	F5	34	c.1601G>A (p.Arg534Gln)	34

[1] NO = Number of, [2]AR = autosomal recessive, [3]AD = autosomal DOMINANT, * = Stop gain, fs = frameshift, § = Low penetrance
