# Supplementary material for: The Landscape of Genetic Variation and Disease Risk in Romania: A Single-Center Study of Autosomal Recessive Carrier Frequencies and Molecular Variants
Source: Int J Mol Sci. 2025 Nov 11;26(22):10912. doi: 10.3390/ijms262210912 (PMC12652900; doi:10.3390/ijms262210912)
Supplement: Supplementary file 1 [file ijms-26-10912-s001.zip › Table S2.pdf]

Table A2. Genes with pseudodeficiency alleles identified in our cohort, which were excluded from statistical analysis and reporting.

**Gene**	**Pseudodeficiency alleles**
ARSA	c.*96A>G (Non-coding)
FAH	c.1021C>T (p.Arg341Trp)
GAA	c.2065G>A (p.Glu689Lys)
GALC	c.550C>T (p.Arg184Cys)
	c.742G>A (p.Asp248Asn)
	c.1685T>C (p.Ile562Thr)
IDUA	c.246C>G (p.His82Gln)
