# Supplementary figures and images for: The Landscape of Genetic Variation and Disease Risk in Romania: A Single-Center Study of Autosomal Recessive Carrier Frequencies and Molecular Variants
Source: Int J Mol Sci. 2025 Nov 11;26(22):10912. doi: 10.3390/ijms262210912 (PMC12652900; doi:10.3390/ijms262210912)

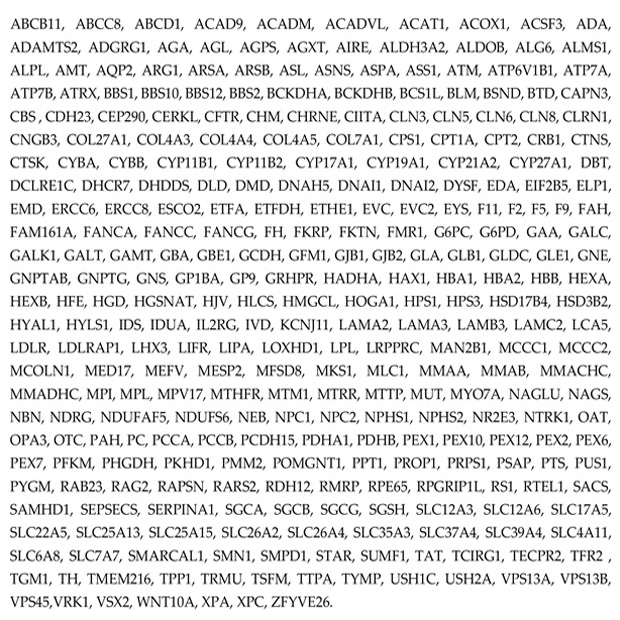

Supplement: Supplementary file 1 [file ijms-26-10912-s001.zip › Figure S1.jpg]
